# Facteurs prédictifs de la ventilation mécanique invasive chez les patients atteints de broncho-pneumopathie chronique obstructive (BPCO)

**DOI:** 10.11604/pamj.2021.39.119.27514

**Published:** 2021-06-11

**Authors:** Lobna Loued, Ahmed Ben Saad, Asma Migaou, Nesrine Fahem, Rania Kaddoussi, Samah Joobeur, Saousen Cheikh Mhamed, Naceur Rouatbi

**Affiliations:** 1 Service de Pneumologie et d´Allergologie, Hôpital Universitaire Fattouma Bourguiba, Monastir, Rue 1er juin, 5000 Monastir, Monastir, Tunisie

**Keywords:** Broncho-pneumopathie chronique obstructive, exacerbation aiguë, facteurs prédictifs, ventilation mécanique, Chronic obstructive pulmonary disease, acute exacerbation, predictive factors, mechanical ventilation

## Abstract

**Introduction:**

le recours à la ventilation mécanique invasive (VMI) au cours des exacerbations aiguës de la broncho-pneumopathie chronique obstructive (EABPCO) constitue un tournant évolutif péjoratif de la maladie. L'intérêt de notre travail est de déterminer les facteurs prédictifs de la VMI lors des EABPCO.

**Méthodes:**

il s´agissait d´une étude rétrospective sur les dossiers des patients hospitalisés dans notre service pour EABPCO durant une période de 18 ans (2000-2017). Nous avons comparé 2 groupes: G1: patients ayant au moins une VMI pour EABPCO et G2: patients qui n´ont jamais eu une VMI suite à une EABPCO.

**Résultats:**

l´étude a inclus 1152 patients BPCO, 133 dans le Groupe G1 (11,5%), et 1019 dans le Groupe G2 (88,5%). Les patients de G1 étaient plus symptomatiques (p < 0,001) avec une obstruction bronchique plus sévère (p < 0,001). Les patients de G1 faisaient plus d´exacerbations (p < 0,001), plus d´hospitalisations et plus de recours à la ventilation non invasive (VNI) (p < 0,001). De même, les patients de G1 développaient plus d´insuffisance respiratoire chronique (IRC) (p < 0,001) et avaient une survie significativement plus basse. Les facteurs de risque indépendants associés à la VMI étaient l´hypercapnie et la baisse du pH lors des EABPCO sévères, l´antécédent de recours à la VNI, et l'état d´IRC.

**Conclusion:**

l´altération de la fonction respiratoire, la sévérité de l´exacerbation, et le recours à la VNI lors d´un épisode antérieur constituent des facteurs prédictifs d´une VMI avec des conséquences péjoratives.

## Abstract (en)

**Introduction:**

the use of invasive mechanical ventilation (IMV) in acute exacerbations of chronic obstructive pulmonary disease (AECOPD) constitutes a negative turning point in the progression of the disease. The purpose of this study is to determine factors predicting the need for IMV in AECOPD.

**Methods:**

we conducted a retrospective study by reviewing the medical records of patients with AECOPD hospitalized in our Department over a 18-year period (2000-2017). We compared 2 groups: G1: patients with AECOPD undergoing at least one IMV and G2: patients who had never undergone IMV following AECOPD.

**Results:**

the study included 1152 patients with COPD: 133 in the G1 group (11.5%), and 1019 in the G2 group (88.5%). G1 patients were more symptomatic (p < 0.001), with more severe bronchial obstruction (p < 0.001). G1 patients had more exacerbations (p < 0.001), more hospitalizations and a higher need for non-invasive ventilation (NIV) (p < 0.001). Similarly, G1 patients more often developed chronic respiratory failure (p < 0.001) and had significantly lower survival rates. Independent risk factors associated with IMV were hypercapnia and decreased pH (in patients with severe AECOPD), a history of NIV, and chronic respiratory failure (CRF).

**Conclusion:**

respiratory function impairment, the severity of exacerbation and the need for NIV in a previous episode are factors predicting the need for IMV and poor outcomes.

## Introduction

La bronchopneumopathie chronique obstructive (BPCO) est une maladie fréquente qui constitue un problème majeur de la santé publique. Elle représente actuellement la troisième cause de décès dans le monde [1]. Le diagnostic repose sur l´existence d´un trouble ventilatoire obstructif défini par un rapport volume expiratoire maximum à la première seconde (VEMS) / capacité vitale forcée (CVF) <70% après administration de bronchodilatateurs [1]. L´évolution de la BPCO est émaillée par des exacerbations aiguës (EABCO) qui sont associées à une lourde morbi-mortalité et à un coût élevé des soins. La mortalité associée aux EABPCO varie de 6% à 32% [2-4].

La ventilation mécanique non invasive (VNI) est le pilier de la prise en charge des patients en EABPCO avec hypercapnie (pCO_2_≥ 45 mmHg) et acidose respiratoire (pH < 7,35) [1]. L´utilisation croissante de la VNI lors des exacerbations aiguës hypercapniques permet de réduire de façon considérable le risque d´une intubation et d´une ventilation mécanique invasive (VMI). Mais malgré le progrès de la VNI, certains patients en EABPCO requièrent une VMI pour maintes raisons, soit d'emblée en cas de détresse neurologique, état de choc ou hypoxémie sévère ou dans un second temps en cas d´échec de la VNI [1]. Les taux rapportés dans la littérature d´échec de la VNI chez les patients en EABPCO varient entre 15 et 24% [5,6]. Chez les patients à haut risque d´échec de la VNI, une intubation précoce est associée à une moindre mortalité hospitalière [7]. De même, une intubation retardée induit plus de complications liées à la ventilation mécanique et majore le taux de décès intra-hospitalier [8]. Par contre, d´autres études déclarent que la VMI constitue en elle-même un facteur de risque d´une mortalité plus élevée chez les patients en EABPCO [9,10].

Les médecins prenant en charge les patients atteints de BPCO en EABPCO sévère et menaçante se trouvent souvent face à un dilemme concernant l´initiation ou non de la VMI. Les opinions des experts varient largement et les guidelines actuelles ne spécifient pas qui sont les patients redevables d´une VMI lors d´une EABPCO [2]. De ce fait, il est nécessaire de déterminer les facteurs prédictifs d´une évolution péjorative d´un patient en EABPCO et d´un besoin d´une VMI, afin de repérer les patients à haut risque et de personnaliser leur suivi et leur prise en charge thérapeutique [4]. L'intérêt de notre travail est de déterminer les facteurs prédictifs de recours à la VMI chez les patients en EABPCO afin d'assurer une meilleure prise en charge de ce groupe de patients à haut risque et de prévenir une évolution péjorative.

## Méthodes

**Type de l´étude**: il s´agit d´une cohorte rétrospective, monocentrique, analytique et comparative, portant sur les dossiers de patients porteurs de BPCO ayant consulté et/ou ayant été hospitalisés au service de Pneumologie au Centre Hospitalo-Universitaire Fattouma Bourguiba de Monastir durant la période allant de janvier 2000 jusqu´à décembre 2017.

### Population cible

**Critères d´inclusion**: les patients inclus dans cette étude sont les malades porteurs de BPCO selon la définition du Global Initiative for Chronic Obstructive Lung Disease (GOLD) qui associe la présence d´une symptomatologie respiratoire chronique faite de toux, expectorations et dyspnée d´effort et la présence d´un trouble ventilatoire obstructif (TVO) à la spirométrie non complètement réversible défini par un rapport volume expiratoire maximum à la première seconde (VEMS)/ Capacité vitale forcée (CVF) < 70% post bronchodilatation [1]. Nous avons défini l´EABPCO selon la définition GOLD comme événement aigu caractérisé par une aggravation des symptômes respiratoires du patient qui sont au-delà des variations quotidiennes normales et conduisent à un changement du traitement [1]. Une exacerbation sévère est définie comme une exacerbation nécessitant l´hospitalisation et exacerbation modérée comme EABPCO nécessitant un traitement antibiotique et/ou courte cure de corticostéroïdes oraux [1]. Une hospitalisation était définie comme un séjour dans un service hospitalier pour une durée supérieure à 24 heures. Après la première hospitalisation, les patients inclus ont eu une période de suivi d'au moins 12 mois. Nous avons calculé pour chaque patient le nombre moyen d'EABPCO (modérés et sévères) par an. Nous avons également considéré les hospitalisations pour EABPCO dans d'autres hôpitaux rapportés par les patients pendant la période de suivi. Nous avons également calculé le nombre moyen d´utilisation de VNI pour EABPCO pour chaque patient et le nombre moyen de recours à la VMI soit d´emblée, soit après échec d´une VNI initiale.

**Critères de non inclusion**: sont exclus de cette étude les patients porteurs d´affections respiratoires chroniques comportant un trouble ventilatoire obstructif permanant et ne rentrant pas dans le cadre des BPCO: l´asthme bronchique dans sa forme chronique avec dyspnée continue, les cas frontières: Asthma COPD Overlap (ACO), les bronchiolites chroniques de l´adulte et certaines formes de bronchiectasies compliquées de trouble ventilatoire obstructif. Nous avons exclus également les patients BPCO admis en réanimation et ayant eu une VMI pour une cause autre que l´exacerbation de BPCO (accident de la voie publique, infarctus du myocarde, etc...).

**Répartition de la population**: les patients ont été répartis en deux groupes: a) Groupe G1: incluant tous les patients BPCO ayant eu recours au moins une fois dans leurs vie à une VMI lors d´une EABPCO d´emblée ou après échec d´une VNI initiale. b) Groupe G2: incluant tous les patients BPCO n´ayant jamais eu une VMI suite à une EABPCO. Nous avons comparé les différents paramètres démographiques, cliniques, biologiques, fonctionnels respiratoires et évolutifs de la BPCO entre les deux groupes.

**La collecte des données**: les différentes variables démographiques (âge, genre, indice de masse corporelle (IMC)), le tabagisme, le grade de dyspnée de base selon la modified Medical Research Council (mMRC), le nombre d´EABPCO par an (EA sévères et modérées), le nombre des hospitalisations pour une EABPCO durant la période du suivi, les explorations fonctionnelles pulmonaires et l´usage d´une oxygénothérapie de longue durée (OLD) sont recueillis à partir des dossiers médicaux. Les spirométries et les gazométries sont faites lors des périodes de stabilité. Le déclin annuel du VEMS est calculé à partir de la pente des équations de régression linéaire chez les patients ayant au moins trois spirométries à l´état stable. Les données concernant la survie sont recueillies à partir des dossiers pour les patients décédés à l´hôpital. Pour les autres patients, les informations sur la survie sont recueillies par contact téléphonique.

**Analyse statistique**: les données ont été saisies et analysées grâce au logiciel SPSS version 18 (Statistical Package for the Social Sciences). Nous avons calculé les fréquences pour les variables qualitatives. Nous avons aussi calculé les moyennes, les médianes, l´écart type (ET) pour les variables quantitatives. Pour déterminer les facteurs associés au recours à la VMI, nous avons comparé les différentes caractéristiques cliniques, paracliniques et évolutives des deux groupes Groupe G1 et Groupe G2, par une étude univariée en utilisant les tests de Khi2, de Fisher et de Student. Le test de Khi2 ou le test de Fisher ont été utilisé pour comparer les variables qualitatives, le test de Student pour comparer les variables quantitatives. Par la suite, nous avons effectué une analyse multi-variée par le modèle de Cox pour identifier les facteurs indépendants prédictifs d´une VMI. Le modèle a inclus toutes les variables significativement associées avec la VMI avec une valeur p < 0,2 en analyse univariée. Les résultats sont exprimés en odd ratio (OR avec un intervalle de confiance (CI) de 95%. La probabilité de survie a été étudiée par la méthode de Kaplan-Meier avec des comparaisons effectuées par les tests de log Rank et Breslow. Le seuil de signification statistique a été fixé à 5%.

## Résultats

Nous avons colligé en tout 1152 dossiers de patients porteurs de BPCO, d'âge moyen de 66 ans. Le nombre moyen d'EABPCO/an était de 2,4±1,7. Durant le suivi, 233 patients (20,2%) ont eu des séances de VNI et 133 patients (11,5%) ont eu une VMI (Tableau 1). Nous avons divisé nos patients en deux sous-groupes, le groupe Groupe G1 comportant les patients qui ont eu au moins une fois dans la vie une ventilation mécanique invasive suite à une EABPCO soit 133 patients (11,5%); et le groupe Groupe G2 comportant les patients BPCO qui n´ont jamais eu une VMI soit 1019 patients (88,5%). Il n´y avait pas de différence entre les deux groupes concernant l´âge, le genre masculin, l´intoxication tabagique, l´indice de masse corporelle (IMC) ainsi que la présence ou non de comorbidités. Par contre, les patients ayant eu une VMI avaient une dyspnée de base plus importante (p<0,001), un trouble obstructif plus sévère (p<0,001), une hypoxémie (p<0,001) et une hypercapnie (p<0,001) plus sévères. De même, les patients ayant une VMI avaient un nombre moyen d´exacerbations aiguës (EABPCO) plus élevé (p<0,001) et des hospitalisations en Pneumologie plus fréquentes (p<0,001).

**Tableau 1:** caractéristiques démographiques des patients hospitalisés pour EABPCO au service de Pneumologie de l´Hôpital Fattouma Bourguiba de Monastir (Tunisie) (N=1152)

Variables	Nombre/ Moyenne	Fréquence
Age(an)	66±10	
Genre(M)	1128	97,9%
Tabagisme(sevré et non sevré)	1142	99%
Consommation tabagique (PA)	59±29	
IMC(kg/m^2^)	24,2±5,5	
Comorbiditiés ≥ 1	911	79%
mMRC≥2	712	61,8%
VEMS (L)	1,26±0,53	
VEMS (%)	45±17	
VEMS/CVF (%)	58±9	
pH	7,40±0,04	
PaO_2_(mmHg)	70±12	
PaCO_2_(mmHg)	40±7	
N EABPCO/an	2,4±1,7	
N H en Pneumologie/an	1,03±0,97	
N H en Réanimation /an	0,21	
N VNI / patient /an	0,21	
N VMI/patient/an	0,16	
IRC	525	45,6%
OLD	170	14,8%
Déclin annuel de VEMS (ml/an)	56,4±31	
Suivi (an)	4,3±3,1	

N: Nombre, M: masculin, PA: paquets-années, IMC : indice de masse corporelle, mMRC : modified Medical Research Council, EABPCO: exacerbation aigue de BPCO, VEMS : Volume expiratoire maximal par seconde, CVF : capacité vitale forcée, H: hospitalisation, VNI : ventilation non invasive, VMI : ventilation mécanique invasive, IRC: insuffisance respiratoire chronique, OLD : oxygénothérapie de longue durée

**Figure 1:**
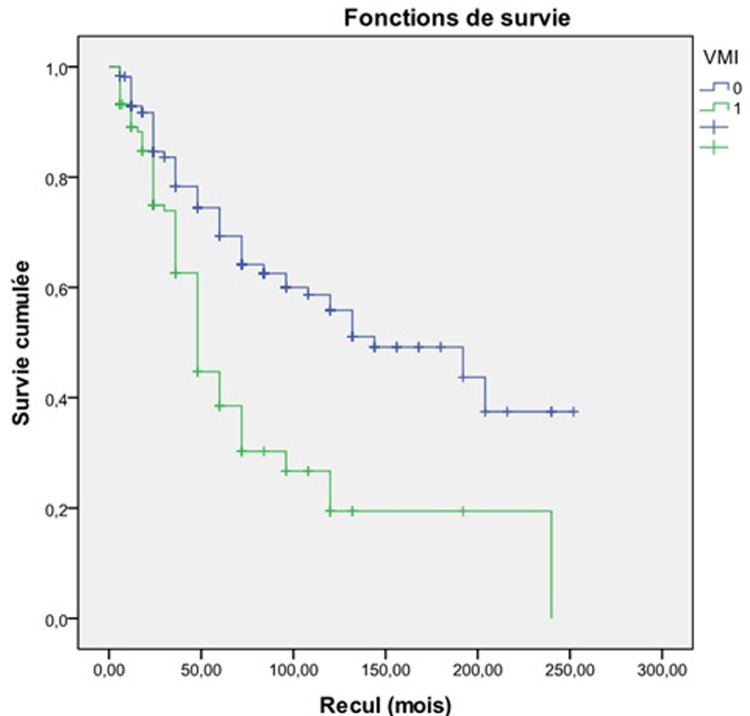
courbe de survie des deux groupes G1 et G2 des patients hospitalisés pour EABPCO au service de Pneumologie de l´hôpital Fattouma Bourguiba de Monastir (Tunisie) (G1: au moins un épisode de ventilation mécanique invasive; G2: pas de ventilation mécanique invasive)

Egalement, le nombre d´épisodes où on a eu recours à la VNI par patient par an était significativement plus élevé chez les patients ayant eu une VMI (p<0,001). Les EABPCO sévères des patients du groupe G1 étaient caractérisées par un pH plus bas (p < 0,001), une PaO_2_ plus basse (< 0,001) une capnie plus élevée (p < 0,001) et plus d´exacerbations dues aux pyocyaniques (p = 0,001). Sur le plan évolutif, les patients du groupe G1 développaient plus d´insuffisance respiratoire chronique (IRC) (p<0,001) et avaient plus recours à une oxygénothérapie au long cours (OLD) (p<0,001) (Tableau 2). L'analyse multivariée avec régression logistique montre qu'une élévation de la capnie lors des EABPCO sévères (p: 0,044), qu´un antécédent de recours à la VNI (p<0,001), qu´une baisse du pH au cours des EABPCO sévères (p: 0,015) et que l'état d´IRC (p<0,001) sont des facteurs de risque indépendants associés à la VMI (Tableau 3). L´étude de la survie a mis en évidence une meilleure survie chez les patients n´ayant jamais eu une VMI (144 mois contre 48 mois; log Rank: <0,001; Breslow: <0,001) (Figure 1).

**Tableau 2:** facteurs associés au recours à la ventilation mécanique invasive chez les patients hospitalisés pour EABPCO au service de Pneumologie de l´hôpital Fattouma Bourguiba de Monastir (Tunisie)

		G1 (N= 133)	G2 (N=1019)	p
Age (an)		67	66,9	0,96
Genre (M)		98,5	97,8	0,46
Tabagisme actif ou sevré		98,5	97,4	0,35
IMC (kg/m^2^)		23,3	24,3	0,07
Comorbidité≥1 (%)		79,7	79,4	0,51
mMRC≥2 (%)		85,7	50,7	**<**0,001
CVF (L)		1,63	2,1	**<**0,001
VEMS après B2 (L)		1,01	1,35	**<**0,001
VEMS/CVF après B2 (%)		54	59	**<**0,001
pH		7,38	7,40	**<**0,001
PaO_2_ (mmHg)		61	71,4	**<**0,001
PaCO_2_ (mmHg)		44,7	39,4	**<**0,001
N EA/an		3,4	2,36	**<**0,001
**Caractéristiques des hospitalisations pour EA**				
	pH	7,36	7,39	**<**0,001
	PaO_2_ (mmHg)	54	60,5	**<**0,001
	Pa CO_2_ (mmHg)	48	41,5	**<**0,001
	CRP (mg/l)	79	82	0,78
	Pyocyanique (%)	11	3,5	**0,001**
NH en Pneumologie /an		1,4	0,84	**<**0,001
VNI (n/patient/an)		1,16	0,23	**<**0,001
IRC (%)		84,2	40,7	**<**0,001
OLD (%)		35,3	12,1	**<**0,001

N: nombre, M: masculin, IMC: indice de masse corporelle, mMRC: modified Medical Research Council, VEMS : Volume expiratoire maximal par seconde, CVF: capacité vitale forcée, N: Nombre, EA: exacerbation aiguë, H: hospitalisation, CRP: C Reactive Protein, VNI: ventilation non invasive, IRC: insuffisance respiratoire chronique, OLD : oxygénothérapie de longue durée

**Tableau 3:** facteurs prédictifs indépendants de la ventilation mécanique invasive chez les patients hospitalisés pour EABPCO au service de Pneumologie de l´hôpital Fattouma Bourguiba de Monastir (Tunisie)

Variables	p	OR	CI (95%)
PaCO2	0,044	1,032	1,001-1,064
Recours à la VNI	<10^-3^	1,472	1,232-1,758
pH EA sévère	0,015	0,004	0,001-0,343
IRC	<10^-3^	2,880	1,627-4,887

VNI: ventilation non invasive, EA: exacerbation aigue, IRC: insuffisance respiratoire chronique

## Discussion

La bronchopneumopathie chronique obstructive (BPCO) est une pathologie inflammatoire respiratoire avec un retentissement systémique, responsable d´une morbidité et d´une mortalité importante [1]. Les exacerbations sévères de BPCO nécessitant une VMI représentent un facteur de gravité et menacent le pronostic vital à court et à long terme. Plusieurs études se sont intéressées à déterminer les facteurs prédisant le recours à la VMI mais leurs résultats étaient hétérogènes. Actuellement, il n´y a pas de recommandations claires dictant quels patients devraient bénéficier d´une VMI. L'objectif de notre étude était de déterminer les facteurs prédictifs de recours à la VMI lors des exacerbations sévères chez les patients du centre Tunisien atteints de BPCO afin d'assurer une détection précoce de ce groupe de patients à haut risque. Ceci va nous permettre de comprendre la réalité évolutive de nos patients, et de personnaliser leur suivi et leur prise en charge. Il s´agissait d´une étude rétrospective portant sur les dossiers de 1152 patients porteurs de BPCO. Les résultats de notre étude suggèrent que le fait d´être en état d´insuffisance respiratoire chronique, qu´un antécédent de recours à la VNI, que l´élévation de la capnie et la baisse du pH lors d´une exacerbation aiguë sévère sont des facteurs de risque indépendants associés au recours à la VMI.

La moyenne d´âge de nos patients était de 66 ans, cette moyenne est comparable avec la littérature décrivant des études similaires [4,11-14] où la moyenne d´âge était comprise entre 59 et 68 ans. Ceci peut être expliqué en partie par le rôle du vieillissement pulmonaire et du tabagisme cumulatif dans la physio-pathogénie de la BPCO [15]. Une nette prédominance masculine (97,9%) a été trouvée dans notre étude, ceci est dû à un tabagisme féminin moindre dans notre pays ainsi que les pays de proximité comme l´Egypte (genre masculin=70%) [12]. Cette différence diminue nettement dans les pays occidentaux où le tabagisme féminin est développé (genre masculin = 47%) [11]. La prévalence de la VMI lors des EABPCO rapportée dans la littérature est très hétérogène, et elle varie de 9,8% à 84,1% [4,12,13,16]. Dans notre étude, le taux de recours à la VMI est de 11,6%. Cette variabilité dépend des critères d´inclusion des patients dans les différentes études ainsi que les conditions des structures sanitaires au sein desquelles ils étaient pris en charge. Dans notre étude, la survie était moindre chez les patients ayant eu une VMI. Nos résultats sont en concordance avec ceux de la littérature [9,10]. En effet, dans l´étude de Alaithan *et al*. [9] concernant l´issue des patients BPCO hospitalisés en unité de soins intensifs, une intubation prolongée a été corrélée à une mortalité plus importante. De même, dans l´étude de Chandra *et al*. [10], les patients ayant une transition de la VNI initiale vers une intubation et une VMI avaient un risque de mortalité multiplié par 6,7 par rapport à ceux placés sur VNI seulement.

Dans l´étude de Khilnani *et al*.les patients ayant eu une VMI avaient un âge plus avancé [14]. Mais dans notre étude, ainsi que dans d´autres études similaires, l´âge n´apparait pas comme un facteur prédictif de recours à la VMI [4,12,13]. Par contre, plusieurs études ont identifié l´âge comme un facteur prédictif de mortalité chez les patients ayant une VMI [17-19]. Le genre, l´intoxication tabagique, l´IMC ainsi que la présence de comorbidités n´étaient pas différents entre les patients nécessitant une intubation et ceux n´ayant pas eu de VMI. Par contre, Madkour et Adly [12] ont décrit que la durée et la quantité du tabagisme en Paquets-années (PA) sont des prédicteurs indépendants de VMI. Ucgun *et al*. [4] ont trouvé sur une analyse multivariée que les patients ventilés mécaniquement et ayant une comorbidité (en particulier l'insuffisance cardiaque congestive) avaient des pires résultats concernant l´évolution et la survie, tandis que les patients sans comorbidités avaient montré des meilleures résultats. Plusieurs études se sont intéressées au rôle des paramètres fonctionnels sur le besoin d´une VMI. Dans une étude de Menzies *et al*. [20], l'état fonctionnel à l´état stable et loin des exacerbations était plus fortement associé au pronostic que toute mesure prise au cours de l´épisode aiguë. Selon eux, cela peut refléter les limites de mesures physiologiques lors de l´épisode aiguë pour évaluer l'impact de maladie pleinement. Dans l´étude de Kumar *et al*. [13], l´état fonctionnel prémorbide était un facteur indépendant de VMI. De même, Vitacca *et al*. [21] ont trouvé que les paramètres spirométriques prédisent le besoin d´une VMI chez les patients présentant une EABPCO. L'effet connu du tabagisme et des exacerbations sévères sur les paramètres spirométriques des patients atteints de BPCO permet d´expliquer que ces derniers présentent un facteur prédictif de VMI [22]. Toutes ces études rejoignent nos résultats qui disent que le groupe des patients nécessitant une VMI avaient une obstruction bronchique plus sévère.

L´élévation de la PaCO_2_ et la baisse du pH étaient toutes les deux des facteurs indépendants prédictifs d´une intubation dans notre étude. Plusieurs études ont signalé que le pH artériel était un facteur indépendant de recours à la VMI. Certains auteurs [4,12-14,23] ont trouvé une association entre l´aggravation de l´acidémie et le taux plus élevé des intubations et ils ont également décrit un pH seuil au-dessous duquel le taux d´intubation était significativement plus élevé chez les patients en IRA secondaire à une EABPCO. Dans l´étude de Kumar *et al*. un pH inférieur à 7,28 était un facteur prédictif indépendant de VMI [13]. Khilnani *et al*. [14] ont identifié le seuil de 7,26 comme un facteur prédictif indépendant de la VMI. Hoo *et al*. [23] ont trouvé que le taux d'intubation était le plus élevé chez les patients ayant un pH <7,20. Ils ont également noté que le temps entre l´arrivée à l´hôpital et l´intubation était plus court lorsque l´acidémie était plus profonde. La PaCO_2_ s'est également avérée significativement plus élevé chez les patients nécessitant une VMI dans une étude de Vitacca *et al*. [21]. Certaines études ont essayé de formuler un score prédictif de mortalité et de ventilation mécanique en vue de stratifier le risque d´une telle évolution. Madkour et Adly [12] ont mené une étude prospective incluant 30 patients dont 56,7% ont eu une VMI. La mortalité intra hospitalière était 13,3%. Les patients sous VMI avaient une exacerbation plus sévère, un état de sepsis, un tabagisme plus lourd, une durée d´hospitalisation plus longue, un pH artériel et un taux de bicarbonate plus bas et un score SAPS II (Simplified Acute Physiology Score) plus élevé. Tous ces éléments ont été inclus dans une équation prédictive. La courbe ROC a montré que l´équation prédictive avait une valeur prédictive modérée avec une sensibilité, spécificité, valeur prédictive positive et négative pour le besoin d´une VMI de 76%, 92%, 93% et 75%, respectivement [12].

Les points forts de notre étude sont la taille importante de l´échantillon, le délai du suivi intéressant et la disponibilité des données épidémiologiques cliniques et fonctionnelles permettant d´étudier de façon précise la sévérité de la maladie et de tracer en temps réel le fil évolutif. D´autre part, la nature rétrospective de notre étude nous a permis d´éviter les biais de sélection inhérent à la sévérité de la BPCO et à l´indication des hospitalisations en premier lieu. De plus, notre travail a été conduit au sein d´un centre hospitalo-universitaire doté d´un service de réanimation polyvalente ce qui a permis de déterminer la prévalence précise de recours à la ventilation mécanique invasive ou non invasive ainsi que l´issue à court et à long termes. Mais le caractère rétrospectif de ce travail garde des limites. Le recueil de certaines données cliniques et para cliniques reste parfois difficile. En effet, il existe un nombre d´exacerbations méconnues qui passent inaperçues et qui ne sont pas signalées par les patients au cours des consultations. D´autre part, des informations importantes manquent de notre études, tels que les scores prédictifs de mortalité comme le score *APACHE II* (Acute Physiology And Chronic Health Evaluation) et le score SAPS II, les paramètres ventilatoires utilisés, la durée d´hospitalisation en réanimation et la mortalité en réanimation. L´idéal serait de réaliser des études prospectives prolongées prenant en compte une population large de patients BPCO et étudiant les paramètres cliniques et biologiques dès leur admission pour exacerbation sévère, jusqu´à l´aggravation et le recours à la VMI. D´autres facteurs prédictifs d´une VMI peuvent ainsi apparaitre, permettant une meilleure sélection d´un groupe de patients vulnérables et une codification particulière de leur suivi et leur prise en charge.

## Conclusion

L´altération de la fonction respiratoire, la sévérité de l´exacerbation avec acidose hypercapnique, les exacerbations fréquentes et le recours à la VNI lors d´un épisode antérieur constituent des facteurs prédictifs d´une ventilation mécanique invasive. Cette dernière s´associe à des conséquences évolutives péjoratives avec une survenue plus élevée d´IRC et une réduction de la survie globale.

### Etat des connaissances sur le sujet

- La bronchopneumopathie chronique obstructive (BPCO) est une maladie fréquente;
- Le recours à la ventilation mécanique invasive (VMI) au cours des exacerbations aiguës de la BPCO (EABPCO) constitue un tournant évolutif péjoratif de la maladie.

### Contribution de notre étude à la connaissance

- L´altération de la fonction respiratoire, la sévérité de l´exacerbation, et le recours à la VNI lors d´un épisode antérieur constituent des facteurs prédictifs indépendants d´une VMI avec des conséquences péjoratives;
- Sur le plan évolutif, les patients ayant eu une VMI développent plus d´insuffisance respiratoire chronique (IRC), ont plus recours à une oxygénothérapie au long cours (OLD) et ont une survie moindre.

## Remerciements

Nous tenons à remercier tout le personnel hospitalier qui a bien renseigné et bien archivé les dossiers que nous avons utilisés pour cette étude.
